# Visual Diagnostics of Dental Caries through Deep Learning of Non-Standardised Photographs Using a Hybrid YOLO Ensemble and Transfer Learning Model

**DOI:** 10.3390/ijerph20075351

**Published:** 2023-03-31

**Authors:** Abu Tareq, Mohammad Imtiaz Faisal, Md. Shahidul Islam, Nafisa Shamim Rafa, Tashin Chowdhury, Saif Ahmed, Taseef Hasan Farook, Nabeel Mohammed, James Dudley

**Affiliations:** 1 Department of Electrical and Computer Engineering, North South University, Dhaka 1229, Bangladeshsaif.ahmed02@northsouth.edu (S.A.);; 2 Adelaide Dental School, The University of Adelaide, Adelaide, SA 5005, Australia

**Keywords:** cariology, deep learning, model ensemble, object detection, transfer learning

## Abstract

Background: Access to oral healthcare is not uniform globally, particularly in rural areas with limited resources, which limits the potential of automated diagnostics and advanced tele-dentistry applications. The use of digital caries detection and progression monitoring through photographic communication, is influenced by multiple variables that are difficult to standardize in such settings. The objective of this study was to develop a novel and cost-effective virtual computer vision AI system to predict dental cavitations from non-standardised photographs with reasonable clinical accuracy. Methods: A set of 1703 augmented images was obtained from 233 de-identified teeth specimens. Images were acquired using a consumer smartphone, without any standardised apparatus applied. The study utilised state-of-the-art ensemble modeling, test-time augmentation, and transfer learning processes. The “you only look once” algorithm (YOLO) derivatives, v5s, v5m, v5l, and v5x, were independently evaluated, and an ensemble of the best results was augmented, and transfer learned with ResNet50, ResNet101, VGG16, AlexNet, and DenseNet. The outcomes were evaluated using precision, recall, and mean average precision (*mAP*). Results: The YOLO model ensemble achieved a mean average precision (*mAP*) of 0.732, an accuracy of 0.789, and a recall of 0.701. When transferred to VGG16, the final model demonstrated a diagnostic accuracy of 86.96%, precision of 0.89, and recall of 0.88. This surpassed all other base methods of object detection from free-hand non-standardised smartphone photographs. Conclusion: A virtual computer vision AI system, blending a model ensemble, test-time augmentation, and transferred deep learning processes, was developed to predict dental cavitations from non-standardised photographs with reasonable clinical accuracy. This model can improve access to oral healthcare in rural areas with limited resources, and has the potential to aid in automated diagnostics and advanced tele-dentistry applications.

## 1. Introduction

Oral health conditions, such as dental caries and its sequeales, are common ailments, aggravated by conditions such as poverty or unsanitary habits, yet only 4.6% of global medical spending is anticipated to go towards oral healthcare [1]. Dental caries is the pathological breakdown of tooth tissue due to a shift in microbiological flora in the oral cavity and an increased secretion of acid. Caries formation is affected by a host of preferential habits, systemic disorders, and congenital anomalies. Incipient carious lesions are often ignored by the patient and treated conservatively by the practitioner, following minimally invasive dentistry protocols. As such, the rates of misdiagnosis and mismanagement are also very high, especially among young practitioners performing visual and radiographic investigations. In rural economies that have limited access to advanced dental infrastructure and experienced practitioners, caries mismanagement can be costly and might leave the patient vulnerable to future periapical, osseus, and fascial space spread of the infection [2]. These issues can be resolved with early detection and regular monitoring via an automated low-cost system that does not discriminate between patients based on their sociodemographic standing in society. The use of mobile handheld devices, such as smartphones, has seen exponential growth in emerging economies, as smartphone ownership and network connectivity has substantially increased within the rural population [3]. As such, recent biomedical research has begun utilising the full functionalities of the sensors in the said devices, to provide affordable solutions to complex problems in remote dental healthcare [4,5,6,7].

Most clinical research that utilises photographs, adheres to strict standardisation protocols, to ensure minimal variations. Strict omission of variables however, translates to a less than optimal performance of the exact same research, when applied in the real world [8]. The current efforts to bridge geographic inequalities in dental diagnostics, will require a robust and adaptive approach, acknowledging that most users in technologically disadvantaged rural regions may not have the skills or expertise to standardise the images captured for remote or automated diagnostics [9]. This is in addition to the ambient light variations present in different regions and at different times of the day, and the masking filters baked into smartphone camera software, that sometimes do a poor job of representing the true colours of an image [10].

Computer vision systems primarily detect regions of interest from multimedia, and have seen a rapid growth in care diagnostics over the last decade. The YOLO (you only look once) system, is a state-of-the-art object detection algorithm, recently used in computer vision, a form of real-time artificial intelligence [11]. YOLO uses a single neural network to process the entire image, segmenting it into sections and forecasting bounding boxes and probabilities for each region [12]. Although several algorithms have been applied in caries diagnostics, YOLO has only been reported for caries diagnostics using radiomic data [13]. When using radiomics for caries diagnostics in rural populations, the application of AI introduces the model to specialist interpretation [14], which can be counterintuitive, because the purpose of the model is to aid in areas where specialist consultations are scarce. The creation of a model ensemble, is a technique that employs various computer vision algorithms on a dataset, to isolate the best performing ones, and then combines the top models to form a ‘super predictor algorithm’. Transfer learning, is another technique that creates a framework for an object detection model to use previously acquired knowledge from different datasets to solve machine learning problems in other fields that have similarities with the existing datasets. To the authors’ knowledge, no previous studies have implemented and validated all three methods together, to generate a caries diagnostic model.

To date, image standardisation has been a serious limitation in digital dentistry, owing to geographic variations in ambient light, operator-induced errors, and availability of high-end imaging hardware [10,15]. Said variables can rarely be addressed in rural clinics with limited resources, thereby limiting the possibilities of automated diagnostics and advanced tele-dentistry applications. There is limited research reporting the diagnostic accuracy of AI-based caries detection using free-hand smartphone photography [4]. The objective of the current study was, therefore, to develop and validate a novel and inexpensive system, utilising a model ensemble and transfer learning, that can predict dental cavitations instantaneously from non-standardised photographs, with reasonable clinical accuracy.

## 2. Material and Methods

The current study design adhered to *Nature Medicine*’s minimum information for clinical artificial intelligence modeling (MI-CLAIM) protocol [16].

### 2.1. Data Input and Pre-Processing

The study consisted of 233 de-identified, pre-extracted anterior teeth. The in vitro simulation study was classified as exempt from ethical review by the relevant ethics committees. A smartphone camera system (12 MP, f/1.8, 26 mm (wide), 1/1.76”, 1.8 µm, Dual Pixel PDAF, OIS; Galaxy s20 5G, Samsung Inc., Seoul, Republic of Korea), focused with a 60× fixed focus optical zoom lens (Lens 9595; Yegren Optics Inc., Anyang, China), was used to capture free-hand images of carious lesions, with no ambient light control. To minimise variations that might adversely affect algorithm training, only human anterior teeth specimens exhibiting visible smooth surface caries and cariogenic activity were selected and isolated from de-identified sources. Molars were excluded, due to potential factors such as shadow casts, altered translucency, occult pit and fissure lesions, and occlusal surface morphology.

The dataset was visually categorised into three classes, based on the appearance of the lesions within the photographs, which were a visual adaptation of the ICDAS classification: ‘visible change without cavitation’, ‘visible change with micro cavitation’, and ‘visible change with cavitation’, following the success documented with the method in recent publications [6,17,18]. The images were labelled by three dentists, after physically inspecting each tooth with a loupe and explorer. A blinded inter-rater reliability Chronbach’s analysis, demonstrated α = 0.958 and r = 0.89 ± 0.06. Each image was processed only when a ĸ = 1.00 agreement was achieved on the proposed classification, following an interactive discussion. Of the 233 images, 68 were excluded, due to not clearly meeting any of the three categories, leaving 165 images for processing. The dataset was split randomly into the following three sets: training data, which consisted of 65% of the images; validation data, which had 15%; and final testing data, which had 20%. While the dataset was considered very small for deep learning, the goal was to create a fully functional model with the least amount of workable data, such that an AI model could be generated that was not primarily limited by the dataset.

### 2.2. Data Augmentation

**Figure 1:**
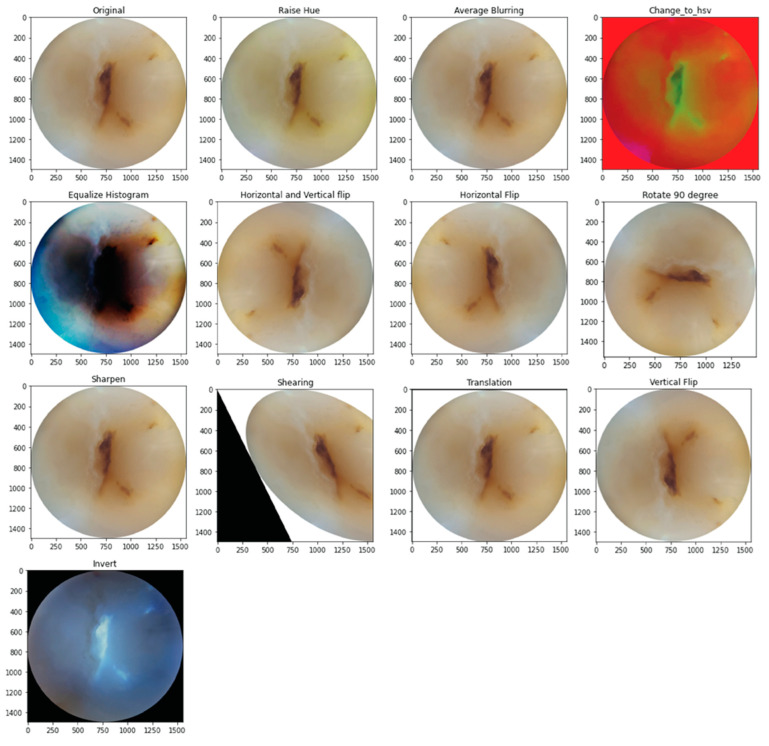
Examples of image augmentation.

The training and validation sets underwent augmentation by 13 different methods. For this purpose, CLoDSA (cross-language object detection and segmentation augmentation) [19], a Python library, was used to simulate real-world variations in the image capture process, including blurry images, images out of focus, incorrect angulation, over- or under-sharpened images, abnormal ambient light filters, etc. [20]. This increased the sample size to 1703 images following augmentation. Figure 1 shows the 13 methods of image augmentation.

**Figure 2:**
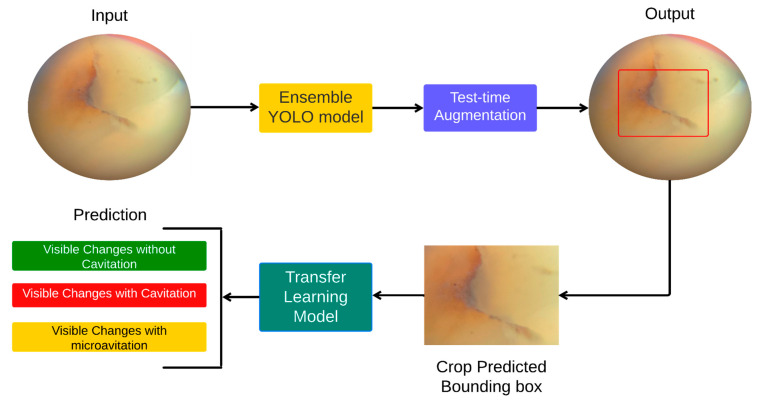
Flowchart summary of the proposed method.

Following data pre-processing and augmentation, a hybrid pipeline for predicting carious lesions using smartphone images of teeth was validated. The pipeline consisted of two steps, namely (1) object detection using a model ensemble and test-time augmentation, followed by (2) transfer learning of the model ensemble. Figure 2 demonstrates the flow chart of the method applied.

### 2.3. Object Detection

**Figure 3:**
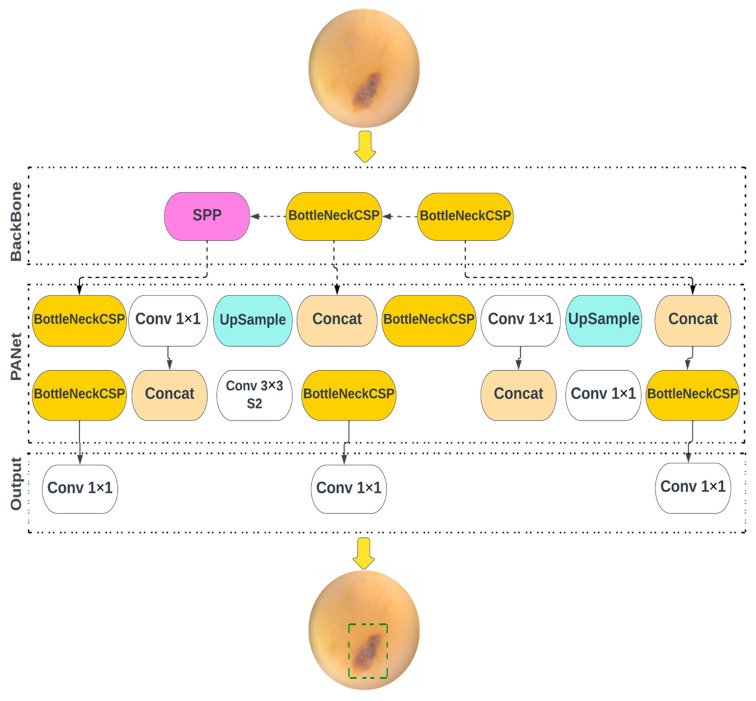
Overview of the object detection architecture deployed for caries diagnostics.

YOLO v5, v5n, v5s, v5m, and v5l models of object detection algorithms were redesigned for use in the current study. The YOLOv5 family of models differ in size and number of parameters. YOLOv5n is the smallest, being less than 2.5 MB in INT8 format and roughly 4 MB in FP32 format, designed for use in edge and IoT devices. YOLOv5s has approximately 7.2 million parameters, and is effective at executing inference on the CPU. YOLOv5m is a medium-sized model, with 21.2 million parameters, and is considered the most appropriate for dataset training, due to its balance of speed and accuracy. YOLOv5l is a large-sized model, with 46.5 million parameters, and is useful for detecting smaller objects. YOLOv5x is the largest, with 86.7 million parameters, and has the highest *mAP*, despite being slower than the others. The models use CSPDarknet as the framework to extract features from images created using cross-stage partial networks, and use binary cross-entropy and the logit loss function, to determine the loss of the trained model, by comparing the target and predicted output values. Figure 3 shows the architecture of YOLO v5. The neck of the models use a feature pyramid network developed by PANet, to combine the features and transmit them to the head for prediction. The YOLOv5 head uses layers to produce predictions from a set of predefined bounding boxes of a certain height and width, also known as anchor boxes.

### 2.4. Ensemble Modelling

Ensemble modelling is a process in which numerous different models are developed to predict a result, either by employing various statistical modelling techniques or by using a variety of training datasets. The approach then combines each base model’s predictions, yielding a single final prediction for the unseen data.

### 2.5. Test-Time Augmentation

Test-time augmentation (TTA) is a technique used to improve the performance of object detection models, by applying various modifications to the test images during the testing phase. The goal of TTA is to improve the robustness of the model, by making it more resistant to variations in the input data. The process generates numerous enhanced copies of each picture in the test set, having the model predict each, and then returning an aggregate of those predictions. In this study, test-time augmentation was applied to all YOLO models to compare the outcomes of the TTA approach with the non-TTA method. In our study, the TTA approach increased the performance of all YOLO models.

### 2.6. Transfer Learning

The images were pre-processed prior to transfer learning. All images, after the augmentation, were cropped and labeled according to their classes and set. A comparative analysis, following different transfer learning models, was performed, based on performance metrics (namely accuracy, precision, recall, and F1 score). Transfer learning models such as ResNet-101, ResNet-50, VGG16, AlexNet, and DenseNet-121 were used for the purpose [21,22,23,24]. ResNet-50 and ResNet-101 are both convolutional neural networks, designed for image classification, with ResNet-50 having 50 layers and ResNet-101 having 101 layers. Both models use skip connections to improve the flow of information between layers, and ResNet101 is pretrained to recognise 1000 different object categories. VGG16, a model developed by the Visual Geometry Group, has 16 weighted layers, including 13 convolutional layers, 5 max pooling layers, and 3 dense layers, and contains a total of 138 million parameters. AlexNet, a groundbreaking model in deep learning for image classification, has eight weighted layers, with the first five being convolutional and the last three being fully connected. It outputs a distribution over 1000 class labels using a 1000-way softmax. DenseNet is another neural network designed for visual object detection, that is similar to ResNet but with some notable differences. It reduces the number of parameters, while improving feature propagation and reuse, and solves the vanishing gradient problem [21,22,23,24].

### 2.7. The Experimental Setup

All hyperparameters and settings were set to ensure uniformity throughout the tests when evaluating the performance of each object detection and transfer learning model. For the object detection model, YOLOv5 has around 30 hyperparameters, that are utilised for various training settings. We utilised the default parameters. During model training, the optimiser of choice was “SGD”, with a learning rate of 10^−2^. The batch size was set to 16, and the number of training epochs was set to 50. Every model was trained on a free GPU from Google Colab on cloud computing, and therefore a learning cost analysis was not performed. The same parameters were used for the transfer learning model training. The optimiser was “SGD”, with a learning rate of 10^−3^. The batch size and epochs were the same as what was used in the object detection model training. ‘Cross entropy loss’ was used as a loss function, with a decay in learning rate of 0.1 every seven epochs.

### 2.8. Evaluation Metrics

Accuracy, precision, recall, F1 score, and mean average precision (*mAP*) were analysed using a confusion matrix, to evaluate the performance of the models. Mean average precision (*mAP*) is a commonly used metric in computer vision, for evaluating the performance of object detection models. The metric is calculated by comparing predicted bounding boxes to ground truth boxes. A bounding box is considered correct if the intersection over union (IoU) between the predicted and ground truth boxes is above a certain threshold. IoU is a metric that measures the degree of overlap between the predicted and ground truth boxes. To calculate *mAP*, precision and recall are computed for various IoU thresholds, and a precision–recall curve is plotted. The average precision (*AP*) for each class, is calculated by finding the area under the precision–recall curve for that class. Finally, the *mAP* is calculated as the average of the AP for all classes. The value of *mAP* ranges from 0 to 1, where higher values indicate better performance. An ideal object detection model would have a *mAP* of 1, while a model that fails to detect any objects would have a *mAP* of 0.

$$mAP=\frac{1}{n}\overset{k=n}{\underset{k=1}{\sum }}AP_{k}$$

$$AP_{k}=the\;AP\;of\;classk$$

n=the number of classes

## 3. Results

### 3.1. Model Ensemble and Test-Time Augmentation

YOLO v5x and YOLO v5l achieved the highest independent mean average precision (*mAP*) values. Table 1 documents the results of the individual YOLO network models, in diagnosing carious lesions. Precision was the highest (0.853) when the YOLOv5l model was used. YOLOv5l and YOLOv5m had the highest overall results of all metrics, thus an ensemble of YOLOv5l and YOLOv5m was further augmented, producing the highest recall (0.705) and *mAP* (0.717) values. Table 2 displays the results of the test-time augmentation for the YOLO network models.

### 3.2. Transfer Learning

Transfer learning models outperformed the base YOLO networks, in terms of precision and recall, across all classifications. The class ‘visible changes without cavitation’, deemed the most challenging to learn, saw an improvement in maximum precision from 0.53 with base YOLO, to 0.76 on a transfer-learned YOLO model. When the VGG16 model was used, the precision (0.89), recall (0.88), and F1 (0.88) scores were the highest. The application of transfer learning on a model ensemble, yielded a diagnostic accuracy of 86.96% on non-standardised free-hand images. The performance outcomes of the transfer learning models on a test dataset, are shown in Table 3.

## 4. Discussion

The current study aimed to develop an inexpensive automation system, to predict dental cavitations instantaneously from non-standardised microphotographs, with reasonable clinical accuracy. For the purpose, the two top YOLO object detection algorithms, with the highest mean average precision (*mAP*), were used, following test-time augmentation and transfer learning. Mean average precision (*mAP*) is a metric used to evaluate the performance of computer vision object detection models, that measures the model’s ability to correctly identify and locate objects within an image by considering both precision and recall, while subsequently highlighting possible areas for improvement. The implementation of data augmentation was performed with careful consideration. Augmentation in medical machine learning, ensures that existing data is transformed by incorporating real-world variations [20,25,26]. Zones of carious decay in the current dataset, were digitally augmented to different inclinations and blurs, to simulate images taken by someone with a hand–brain coordination disorder or someone who may not be very well versed with smart devices, such as those in rural outreaches of developing countries that have only recently opened up to the technology [9,27]. The elaborate method of data augmentation was complimented by test-time augmentation. Test-time augmentation (TTA) is a novel approach to caries detection. In the current research, TTA served to provide the model with a ‘reality check’, by applying various modifications to the images during testing, making the model more robust and versatile, improving its ability to handle real-world variations.

Ironically, the core foundation of automated caries detection from real-world variations was built on previously established research on mathematical approaches that generated ‘rule-based’ AI models [28]. These rule-driven models were always more efficient in diagnosing caries than human practitioners [28,29]. Data collected from interviews, to train these models, have a relatively predictable set of variations [30]. Automated classification of carious involvement from radiographs or thermal imaging, by comparison, is an easier task, as the core principle is to differentiate across pixels of radiolucency and opacities, or changes in Fourier or wavelet-based features, on a relatively standardised imaging modality [31,32]. This held true when radiographic data was collected from 100 clinics and the AI model was able to successfully classify all the lesions [31]. In both cases, the sensitivity and specificity were above 90%. If oral photographs were collected as data from 100 clinics, there would likely be over a thousand variations from a lack of image standardisation alone [15].

Radiographic image processing through older methods, such as support vector machine (SVM), back-propagation neural networks (BPNN), and fast convolutional neural networks (FCNN), have also been documented in the past, with approximately 3% variations in classification accuracy across the models, yet were still 10% more accurate and consistent than dental practitioners in classifying carious lesions [33,34]. The use of ICDAS [35] for visual classification of carious lesions from photographs, generates mixed opinions, as experts argue that caries classifications should be based on the depth of the lesion, and a 2-dimensional image may be inadequate in determining the actual extent, without supporting images of histologically cross-sectioned teeth [18]. Yet, the use of such invasive methods in vivo are rightfully contraindicated in patient care, and the use of ICDAS caries classification models remains very popular in carious image recognition in SVM, BPNN, and FCNN, with studies of 500+ intraoral images yielding overall accuracies of 80 to 90% without histological cross-sectioning [18,36]. The current study also applied the ICDAS classification, but on a comparatively smaller dataset of lesions, where the ensemble and VGG16 transfer-learned model performed as efficiently and more reliably than previously documented classification models such as SVM, BPNN, FCNN.

Many dental researchers have transitioned to YOLO-based object detection, to automate carious lesion detection. Sonavane et al. found YOLO to successfully classify carious lesions at 87% accuracy, when the threshold for positivity was leniently set at a cutoff value of 0.3 [37]. The current study stressed the models further, by setting a cutoff value at 0.5, ensuring higher scrutiny. Diagnosing visual changes that have not cavitated, is a clinical challenge in minimally invasive dentistry and atraumatic restorative treatment (ART) procedures [38]. Thanh et al. implemented a mobile phone-based diagnostic tool for self-reporting of carious lesions. Similar to the current study, the authors classified smooth surface caries, as they were deemed the most challenging to diagnose, and implemented different deep learning models [39]. Even for the top performing models, YOLO v3 and FRCNN, that produced overall accuracies of 71.4 and 87.4%, respectively, the diagnostic sensitivity in detecting non-cavitated lesions was only 36.9% and 26%, respectively [6]. Application of an ensemble YOLO model and transfer learning in the current study, was able to improve the outcomes drastically, to over 85%. In contrast to existing work performed on caries detection [13], the current study focuses on images taken from handheld devices, with the justification that underdeveloped regions of emerging economies may not have access to professional imaging equipment. The current study also implemented a blend of object detection classifiers, a model ensemble, test-time augmentation, and multiple transfer learning classifiers, and statistical evaluation through mean average precision, all of which have not been performed in any previous reports documenting AI application in caries diagnostics [13].

### Limitations

Models of carious lesion detection rely on heavy computational hardware. Recent documentation of 8554 carious images’ diagnostics, using transformer mechanisms on a RDFNet architecture during feature extraction, had to be trained on very high-end machines, running on an NVIDIA GeForce RTX 3090 graphics card, with 24 GB of RAM, using the Pytorch deep-learning framework [40]. The current study was directed by previous reports of caries diagnostics, that recommended 103 to 585 images to be appropriate [18,36,41]. However, a formal power analysis was not performed in the current study, which could serve as a limitation. The current study was also performed using cloud computing (Colaboratory; Google Inc., Mountain View, CA, USA), which can serve as both an advantage and limitation. The advantage is that the cost of hardware procurement is avoided, making the model feasible for development using data obtained in less resource-rich communities. Such an installation, however, is predominantly dictated by the network’s bandwidth, through which they are transmitted, and fluctuations in network strength can significantly delay inference time, thereby introducing noticeable latency in real-time diagnostics. A cost sensitive analysis [42] could not be performed on the model for the very same reasons. Due to the absence of discernible differences in enamel and dentin caries within the available dataset, an estimation of caries depth could not be performed.

Future research should include working on greater variations in the carious dataset, cross-sectional imaging for caries depth analyses, the application of multi-label classifications [43], processing the data through Explainable AI [44], and the installation of the trained models into smart glasses, to pilot a single centre patient population.

## 5. Conclusions

Within the limitations of the current in vitro simulation, it can be concluded that:

1. An ensemble model, created using various YOLO computer vision models, and transferred to VGG16 methods of deep learning, can generate accurate predictions in diagnosing smooth surface caries from free-hand photography.
2. Ensembles of computer vision algorithms, that undergo augmentation and transfer learning, can lead to the formation of inexpensive digital diagnostic markers, that practitioners can use to screen and monitor progression of carious lesions.

## Figures and Tables

**Table 1:** Results obtained following model ensemble for YOLO network.

Model	Classification	Precision	Recall	Test Map@0.5
YOLO v5n	Visible change without cavitation	0.30	0.40	0.28
	Visible change with microcavitation	0.76	0.54	0.65
	Visible change with cavitation	0.72	0.88	0.87
	Overall	0.59	0.60	0.60
YOLO v5s	Visible change without cavitation	0.60	0.54	0.41
	Visible change with microcavitation	0.89	0.64	0.72
	Visible change with cavitation	0.80	0.75	0.86
	Overall	0.76	0.64	0.66
YOLO v5m	Visible change without cavitation	0.55	0.54	0.39
	Visible change with microcavitation	0.99	0.58	0.69
	Visible change with cavitation	0.85	0.75	0.86
	Overall	0.80	0.62	0.65
YOLO v5l	Visible change without cavitation	0.55	0.54	0.56
	Visible change with microcavitation	0.94	0.62	0.74
	Visible change with cavitation	0.91	0.75	0.83
	Overall	0.80	0.64	0.71
YOLO v5x	Visible change without cavitation	0.52	0.43	0.53
	Visible change with microcavitation	0.82	0.58	0.69
	Visible change with cavitation	0.95	0.88	0.92
	Overall	0.76	0.63	0.71

**Table 2:** YOLO network results obtained with test-time augmentation.

Model	Classification	Precision	Recall	Test Map@0.5
YOLO v5n	Visible change without cavitation	0.38	0.46	0.47
	Visible change with microcavitation	0.66	0.58	0.61
	Visible change with cavitation	0.82	0.75	0.84
	Overall	0.62	0.60	0.64
YOLO v5s	Visible change without cavitation	0.60	0.54	0.49
	Visible change with microcavitation	0.77	0.63	0.70
	Visible change with cavitation	0.99	0.75	0.81
	Overall	0.77	0.64	0.67
YOLO v5m	Visible change without cavitation	0.48	0.57	0.50
	Visible change with microcavitation	0.87	0.63	0.72
	Visible change with cavitation	0.83	0.88	0.91
	Overall	0.73	0.69	0.71
YOLO v5l	Visible change without cavitation	0.63	0.52	0.48
	Visible change with microcavitation	0.93	0.71	0.75
	Visible change with cavitation	1.00	0.82	0.89
	Overall	0.85	0.68	0.71
YOLO v5x	Visible change without cavitation	0.54	0.46	0.46
	Visible change with microcavitation	0.83	0.61	0.67
	Visible change with cavitation	0.99	0.75	0.92
	Overall	0.79	0.61	0.68
YOLO model ensemble (v5m + v5l)	Visible change without cavitation	0.51	0.54	0.50
	Visible change with microcavitation	0.94	0.71	0.74
	Visible change with cavitation	0.87	0.87	0.91
	Overall	0.77	0.71	0.72

**Table 3:** Transfer learning model performance result.

Model	Classification	Precision	Recall	F1 Score	Accuracy
VGG16	Visible change without cavitation	0.76	0.93	0.84	
	Visible change with microcavitation	0.91	0.83	0.87	
	Visible change with cavitation	0.99	0.88	0.93	
	Overall	0.89	0.88	0.88	86.96%
Resnet50	Visible change without cavitation	0.64	0.64	0.64	
	Visible change with microcavitation	0.88	0.92	0.90	
	Visible change with cavitation	0.71	0.62	0.67	
	Overall	0.75	0.73	0.74	78.26%
Resnet101	Visible change without cavitation	0.73	0.79	076	
	Visible change with microcavitation	0.88	0.92	0.90	
	Visible change with cavitation	0.99	0.75	0.86	
	Overall	0.87	0.82	0.84	84.78%
Alexnet	Visible change without cavitation	0.68	0.93	0.79	
	Visible change with microcavitation	0.95	0.83	0.89	
	Visible change with cavitation	0.83	0.62	0.71	
	Overall	0.82	0.80	0.80	82.60%
Densenet121	Visible change without cavitation	0.75	0.86	0.80	
	Visible change with microcavitation	0.91	0.88	0.89	
	Visible change with cavitation	0.86	0.75	0.80	
	Overall	0.84	0.83	0.83	84.78%

## Data Availability

The repository https://github.com/Tareq361/Dental-caries-detection-using-a-Hybrid-Ensembled-YOLO-and-Transfer-Learning-Model (accessed on 8 February 2023) contains the codes and additional relevant data related to the current study.
